# Prevalence of Depressive Symptoms and Associated Factors among HIV-Positive Youth Attending ART Follow-Up in Addis Ababa, Ethiopia

**DOI:** 10.1155/2019/4610458

**Published:** 2019-01-02

**Authors:** Helina Abebe, Shegaye Shumet, Zebiba Nassir, Melkamu Agidew, Dessie Abebaw

**Affiliations:** ^1^Menelik II Health Science College, Addis Ababa, Ethiopia; ^2^Department of Psychiatry, University of Gondar, Gondar, Ethiopia; ^3^Amanuel Mental Specialized Hospital, Addis Ababa, Ethiopia; ^4^Department of Epidemiology, Jimma University, Jimma, Ethiopia

## Abstract

Depression is most frequently and highly occurring common mental disorder in HIV/AIDS patients especially youth living with HIV/AIDS. This study aimed to assess the prevalence and associated factors of depressive symptoms among youth living with Human Immunodeficiency Virus (HIV) attending Antiretroviral Therapy (ART) follow-up at public hospitals in Addis Ababa, Ethiopia.* Objective*. To assess the prevalence and associated factors of depressive symptoms among youth living with Human Immunodeficiency Virus (HIV) attending Antiretroviral Therapy (ART) follow-up at public hospitals Addis Ababa, Ethiopia, 2016.* Method*. In a cross sectional study, 507 HIV-positive young people from public health hospitals were recruited by systematic random sampling technique. Beck Depression Inventory-II was used to assess depressive symptoms. Morisky medication adherence rating scale, social support rating scale, and HIV stigma scale were the instruments used to assess the associated factors.* Results*. Prevalence of depressive symptoms among HIV-positive youth was 35.5% (95% CI:31.3, 39.6). In multivariate analysis, age range between 20 and 24 years with (AOR=2.22, 95% CI: 1.33,3.62), history of opportunistic infection (AOR=1.94, 95% CI:1.15,3.27), poor medication adherence (AOR=1.73, 95%CI:1.13,2.64, low social support (AOR=2.74, 95%CI:1.13,2.64), moderate social support (AOR=1.75 95% CI: 1.03,2.98), and stigma (AOR=2.06, 95% CI: 1.35,3.14) were associated with depressive symptoms. The results suggest that prevalence of depressive symptoms among HIV-positive youth was high. Prevention of opportunistic infection, stigma, and counseling for good medication adherence are necessary among HIV-positive youth.

## 1. Introduction

According to DSM-5 depression is a common mental disorder that presents with depressed mood, loss of interest (pleasure), decreased energy, feeling of guilt, or low self-worth, disturbed sleep or appetite, and poor concentration [1]. HIV/AIDS is a chronic infectious disease and first leading cause of mortality and morbidity worldwide from an infectious disease. An estimated 5 million young people aged 15-24 years are living with HIV, vast majority in sub-Saharan Africa [2]. Depressive disorders were the third leading cause of global burden of disease in 2004 and will be the first by 2030. Globally depression is number one cause of illness and disability among youth [3–7].Youth and young adults account for a large percentage of all HIV/AIDS cases in Ethiopia [8]. AIDS death has risen only among adolescents and young age group since 2001 [9, 10]. There is an evidence of significant comorbidity in people living with HIV/AIDS (PLWHA) including depression [4, 5].

Different studies have shown the global magnitude of depression among HIV-positive youth to vary across the world. About 44% in America [11] and in Africa, studies report prevalence to ranges between 12 and 60% [12]. Another studies in Malawi, Malaysia, and Kenya revealed that the prevalence of depression among HIV-positive youth was 18.9%, 18.3%, and 48%, respectively [4, 5, 13].

Mental health and HIV/AIDS are closely interlinked. Living with HIV/AIDS can increase the risk of mental illness such as depression. While poor mental health can inspire behaviors which place individuals at risk for HIV/AIDS [14]. Depression negatively affects HIV disease prognosis. It decreases CD4 T lymphocytes activity, increases viral load, and affects quality of life and medication adherence, which contributes a greater risk of mortality [15].

There are a number of moderating factors for depression in PLWHA. The moderating factors were age, gender, primary care giver type, maternal death, change in care giver type, death in the family [14, 16, 17], failing in the school term, medication adherence, HIV related stigma, and poor social support which were the major moderating factors for depression among HIV-positive youth [4, 18, 19].

Depression in youth has also been correlated with high risk behaviors including earlier sexual debut, low condom use, substance abuse, more frequent sexual partners, and unplanned pregnancy [4, 5].

Youth and young adults account for a large percentage of all HIV/AIDS cases in Ethiopia. Living with HIV/AIDS can increase the risk of mental illness such as depression. Even though neuropsychiatric comorbidities (including depression) are highly prevalent among HIV-positive youth worldwide, there are no studies which show prevalence of depression among HIV-positive youth in Ethiopia. So determining prevalence of depressive symptoms and associated factors among HIV-positive youth is important for early intervention and further decreases the burden of depression and contributes to have a plane for improving patients' quality of life.

## 2. Methods and Materials

### 2.1. Study Settings and Populations

An institution based cross sectional study was conducted in nine hospitals (Black Lion, Ras Desta, St. Peter and Yekatit 12, Gandhi memorial, Minlik II, St. Paulos, Alert, and Zeweditu Memorial Hospital) in Addis Ababa, Ethiopia, between May and June 2016. The study subjects were selected from these public hospitals using systematic random sampling technique for interview.

### 2.2. Measurement

Beck Depression Inventory-II (BDI-II) was used to assess the presence and severity of depressive symptoms. The tool consists of 21 items and patients with a score greater than or equal to 21 [4] were taken as having depressive symptoms. The sociodemographic factors were age, sex educational status, and marital status.

The four-item Morsiky Medication Adherence Rating Scale (MMARS) questionnaire was used to measure drug nonadherence. The cut point of 3 and above was used to define nonadherence [20]. Social support was assessed by adolescent social support rating scale. The scale has three broad categories “low social support” (1-2.9), “moderate social support” (3-5), and “strong social support” (5.1-7) [21]. Stigma was measured by a 12-item HIV stigma scale [22]. The scale consists of four-point Likert scale (strongly disagree, disagree, agree, and strongly agree) questions concerning disclosure status, negative self-image, and public attitudes. Data regarding stages of HIV, base line CD4 count, types of ART, and history of opportunistic infection were obtained from patient's medical chart.

### 2.3. Data Collection

Data were collected by four trained data collectors (general nurses) using the Amharic version of the questionnaire for a month. The questionnaire was designed in English and was translated to Amharic and back to English, that is, forward and backward translation. The training was on introduction to depression and HIV comorbidity, research methods, interviewing skills, sampling and recruitment, and ethical aspects of research.

### 2.4. Data Processing and Analysis

All collected data were checked for completeness and consistency and entered in to EPI INFO version 7 and then exported to SPSS for windows version 20 for analysis.

Descriptive and bivariate logistic regression analyses were computed to see frequency distribution and to test whether there was an association between the independent and dependent variables, respectively. Factors associated with depressive symptoms were selected during bivariate analysis with a value of p≤0.2 for further analysis in multivariable regression analysis. Variables with P-value less than 0.05 at 95% confidence interval were considered as statistically significant.

### 2.5. Ethical Consideration

Ethical clearance was obtained from University of Gondar institutional review board. Permission was obtained from Addis Ababa City Administration Health Bureau Ethical Committee. Written consent was taken from study participants and assent from legally approved foster parents after explaining purpose of the study. Confidentiality was maintained by omitting their personal identification.

## 3. Results

A total of 507 participants out of 537 enrolled were included in the study making the response rate 94.4%. The mean age of participants was 18.6 years (±SD =3.024) and 272 (69.6%) were females. Concerning educational status, about 232 (45.8%) had attended primary education. From a total of participants 243 (47.9%) were living with father/mother (Table 1).

Out of the total respondents the majority 280 (75%) were on WHO stage I and II disease and 409 (80.7%) were taking first line ART treatment. About 283 (55.6%) had poor adherence on medications and 261 (51.5%) experienced stigma because of their HIV status (Table 2, see Appendix).

### 3.1. Prevalence of Depressive Symptoms

In our finding the prevalence of depressive symptoms among the study participants was 35.5 % with 95% CI (31.3%, 39.6%).

### 3.2. Factors Associated with Depressive Symptoms among HIV-Positive Youth

Bivariate analysis indicated that sociodemographic variables (age, sex, educational status, primary care giver type, and living status), clinical variables (stage of HIV and history of opportunistic infections), and psychosocial factors (stigma, adherence, social support, and ever use of substance) were significantly associated with depressive symptoms. In the multivariate model age, history of opportunistic infection, experience of stigma, poor medication adherence, and low and moderate social support were statistically significant with depressive symptoms (Table 3, see Appendix).

## 4. Discussion

Prevalence of depressive symptoms among HIV-positive youth was 35.5% with 95% CI (31.3%, 39.6%) based on the Beck inventory scale. The rate is lower than reported rates in Zimbabwe 63% [23], Kenya 48% [24], and USA 52 % [25]. The possible reason for this variation might be due to the difference in study design, sample size, and data collection tools. It might also be related to substance, stigmatization, and low social support. Substance use may increase risk of HIV infection and AIDS and interfere with their treatment, and conversely some mental disorders occur as a direct result of HIV infection. Youth with depression may have low in treatment service for HIV/AIDS. On the other hand, our rates are higher than the study done in Malawi, 18.9% [4] Kenya 17.8% [13], and Malaysia 18.5 % [26]. The possible reason for this discrepancy might also be due to the study design and data collection tools. It might also be due to very poor medication adherence and presence of opportunistic infection in our study.

Our study identified an association between age and depressive symptoms among youth with HIV. Specifically, age range from 20 to 24 years was significantly associated with depressive symptoms. The odds of developing depressive symptoms among participants with age range of 20-24 years were 2.2 (AOR= 2.20, 95% CI: 1.33, 3.62) times higher compared with age range of 15-19 years. As age increases the level of understanding and conceptualizing their HIV status also increases as well as entry to adult hood which may be fraught with developmental challenges. It might be difficult for youth coping with HIV since birth, including ongoing treatment and hospitalization which leads to low self-image. The aforementioned study from Malawi [4] and USA [25] also identified the relationship between increased age and depression.

Respondents who had poor adherence on medication were 1.73 times more likely to develop depressive symptoms than those respondents who had good adherence on medication (AOR =1.73, 95%CI: 1.13, 2.64). This might be due to poor medication adherence which has been implicated in the emergence of drug resistance strains of HIV, which results in decrease in virology suppression. The finding is supported by study in Zimbabwe [11] and Kano, North Western Nigeria [27].

With respect to social support respondents who had moderate social support and low social support were 1.75 and 2.74 times more likely to develop depressive symptoms than those who had strong social support with AOR= 1.75, 95% CI: 1.03, 2.98 and AOR =2.74 95%CI: 1.42, 5.27, respectively. Young people with no close family to disclose their problem are unable to get care and may have increased depressive symptoms [25]. This might also be due to decreased social support within the context of HIV; AIDS is related to increased depression because of various factors such as educational disability, food insecurity, isolation, and debilitation. Low social support could result in poor adherence to medication and as a result poor adherence leads to immune suppression which finally leads to depression [13, 28].

HIV related stigma increases the individual vulnerability 2.06 times in risk of developing depressive symptoms with (AOR=2.06, 95% CI: 1.35, 3.14). Our finding is consistent with the study done in different developed and developing countries [29, 30]. It might be related to the fact that young people with HIV status develop increase in sense of isolation, poor self-worth, and rekindle hopelessness which might lead to psychological distress.

With regard to opportunistic infections, respondents who had history of opportunistic infections were 1.94 times in risk of developing depressive symptoms (AOR=1.94 95% CI:1.15, 3.27). Immunosuppression and dissatisfaction with one's physical appearance, all possibly the results of HIV infection, could result in depression. The aforementioned study done in Malawi also supports the current study [4].

There were several limitations. The design of the study was cross sectional; therefore we were unable to conclude any causal direction of the association found and have no information regarding timing (onset) of depressive symptoms. Many of the variables were self-reported and therefore the respondents may be influenced by social desirability bias.

## 5. Conclusion

HIV-positive young people were at high risk of developing depression. Importantly this study demonstrates a high prevalence of depressive symptoms among HIV-positive youth attending ART follow-up at public hospitals. Age, history of opportunistic infection, HIV related stigma, poor medication adherence, and low and moderate social support were found to be independent predictor of depressive symptoms. Integration of mental health evaluation and treatment into the HIV care provided for youth can be beneficial. More studies to delineate factors associated with depressed youth with HIV may add value to the body of knowledge and overall improvement of care. The limitation of this study was social desirability bias.

## Figures and Tables

**Table 1:** Sociodemographic characteristics of HIV positive youth on ART follow-up at selected public hospitals in Addis Ababa, Ethiopia, 2016 (n=507).

Characteristics	Number	Percent (%)
Age		
15-19	353	69.6
20-24	154	30.4
Sex		
Female	272	53.6
Male	235	46.4
Educational status		
Primary	232	45.8
Secondary	212	41.8
Diploma and above	63	12.4
Primary care giver		
Both mother and father	214	42.2
Other siblings	293	57.8
Living status of mother/father		
Mother/father died	243	47.9
Both died	107	21.1
Both alive	157	31.0

Others = grandmother/father, aunt/uncle, and brother/sister.

**Table 2:** Distribution of clinical, stigma, and psychosocial factors among HIV positive youth attending ART follow-up at public hospitals in Addis Ababa, Ethiopia, 2016(n=507).

Characteristics	Frequency	Percent (%)
Stage of HIV		
Stages 1 and 2	380	75
Stages 3 and 4	127	25
ART type		
First line	409	80.7
Second line	98	19.3
Opportunistic infection		
Yes	162	32
No	345	68
CD4 count		
0-300	206	40.6
>350	301	59.4
Stigma		
Yes	261	51.5
No	246	48.5
Adherence		
Poor	282	55.6
Good	225	44.4
Social support		
Low	92	18.1
Moderate	293	57.8
Strong	122	24.1
Taunt for physical appearance		
Yes	47	9.3
No	460	90.7
Taunt for taking ART		
Yes	15	3.0
No	492	97.0
Substance use		
Yes	152	30
No	355	70

**Table 3:** Factors associated with depression among HIV positive youth attending ART follow-up at public hospitals in Addis Ababa, Ethiopia, 2016 (n= 507).

**Characteristics **		**Depression **		**COR**	**AOR**
		**Yes **	**No **	** 95**%** CI**	** 95**%** CI**
**Age **	15-19	99(28.0)	254(72.0)	1.00	1.00
	20-24	81(52.6)	73(47.4)	**2.84(1.92, 4.21)**	**2.20(1.33, 3.62)** **∗** **∗**
Sex	Female	105(38.6)	167(61.4)	1.34(0.92, 1.93)	1.21(0.79, 1.84)
	Male	75(31.9)	160(68.1)	1.00	1.00
Educational status	Primary	73(31.5)	159(68.5)	0.47(0.26, 0.83)	0.70(0.35, 1.37)
	Secondary	76(35.8)	136(64.2)	0.57(0.32, 1.01)	0.99(0.50, 1.96)
	Diploma and above	31(49.2)	32(50.8)	1.00	1.00
Primary care giver	Other sibling	120(41.0)	173(59.0)	1.78(1.21, 2.59)	0.88(0.53, 1.47)
	Mother/father	60(28.0)	154(72.0)	1.00	1.00
living status of mother /father	Both dead	29(27.1)	78(72.9)	0.62(0.41, 0.93)	0.73(0.36,1.47)
	Mother/father died	81(33.3)	162(66.7)	0.46(0.27, 0.78)	1.04(0.62, 1.75)
	Both alive	70(44.6)	87(55.4)	1.00	1.00
Stages of HIV	Stage 3&4	54(42.5)	73(57.5)	0.67(0.44, 1.01)	1.00(0.55, 1.82)
	Stage 1&2	126(33.2)	254(66.8)	1.00	1.00
**Opportunistic infection**	Yes	74(45.7)	88(54.3)	**1.89(1.29, 2.78)**	**1.94(1.15, 3.27)** **∗** **∗**
	No	106(30.7)	239(69.3)	1.00	1.00
**Stigma **	Yes	116(41.2)	132(52.8)	**2.74(1.88,4.00)**	**2.06(1.35, 3.14)** **∗** **∗** **∗**
	No	64(24.5)	197(75.5)	1.00	1.00
**Adherence **	Poor	121(42.9)	161(57.1)	**2.11(1.44, 3.09)**	**1.73(1.13, 2.64)** **∗** **∗**
	Good	59(26.2)	166(73.8)	1.00	1.00
**Social support **	Low	44(47.8)	48(52.2)	**3.22(1.78, 5.82)**	**2.74(1.42, 5.27)** **∗** **∗**
	Moderate	109(37.2)	184(62.8)	**2.08(1.27, 3.39)**	**1.75(1.03, 2.98)** **∗**
	Strong	27(22.1)	95(77.9)	1.00	1.00
CD4 count	0-350	80(38.8)	201(66.8)	1.27(0.88, 1.84)	0.93(0.57, 1.50)
	>350	100(33.2)	126(61.2)		
Substances	Yes	80(52.6)	72(47.4)	2.83(1.91, 4.19)	1.85(0.68, 4.99)
	No	100(28.2)	255(71.8)	1.00	1.00

. *∗∗∗*  ***indicates p-value<0.001,  *∗∗* indicates p-value<0.01, and  *∗*indicates p-value <0.05.***

## Data Availability

The datasets used for current study are available by asking a reasonable request (helusimren@gmail.com).

## Appendix

See Tables 2 and 3.

## Abbreviation

AH: Alert Hospital
AMSH: Amanuel Mental Specialized Hospital
AIDS: Acquired Immune Deficiency Syndrome
ART: Antiretroviral Therapy
BDI-II: Beck Depression Inventory Version II
DMH: Dagmawi Menelik Hospital
GMH: Gandhi Memorial Hospital
HIV: Human Immune Virus
LMIC: Low and middle income countries
MINI: Mini-International Neuropsychiatric Interview.
